# Correlations between APACHE-II score and pressure parameters of mechanical ventilation in patients with ARDS and their value in prognostic evaluation

**DOI:** 10.12669/pjms.39.6.7190

**Published:** 2023

**Authors:** Wei Lu, Jiansong Zhang, Yao Qiu, Na Fei, Liansen Yin

**Affiliations:** 1 Wei Lu, Intensive Care Unit, Shengzhou Hospital of Traditional Chinese Medicine, Shengzhou 312400, Zhejiang, China; 2 Jiansong Zhang, Intensive Care Unit, Shengzhou Hospital of Traditional Chinese Medicine, Shengzhou 312400, Zhejiang, China; 3 Yao Qiu, Intensive Care Unit, Shengzhou Hospital of Traditional Chinese Medicine, Shengzhou 312400, Zhejiang, China; 4 Na Fei, Intensive Care Unit, Shengzhou Hospital of Traditional Chinese Medicine, Shengzhou 312400, Zhejiang, China; 5 Liansen Yin, Intensive Care Unit, Shengzhou Hospital of Traditional Chinese Medicine, Shengzhou 312400, Zhejiang, China

**Keywords:** Acute respiratory distress syndrome, APACHE-II, Plateau pressure, Driving pressure, Mean airway pressure

## Abstract

**Objective:**

To investigate the correlations between APACHE-II score and pressure parameters of mechanical ventilation in patients with acute respiratory distress syndrome (ARDS) and their value in prognostic evaluation.

**Methods:**

This was a retrospective study. The clinical data of 79 patients with ARDS treated in Shengzhou Hospital of Traditional Chinese Medicine from April 2020 to April 2022 were analyzed retrospectively. According to whether their APACHE-II scores were higher than 15, they were divided into low score group (n= 20) and high score group (n= 59). The plateau pressure (Pplat), driving pressure(ΔP) and mean airway pressure (Pmean) were compared. The correlation between APACHE-II score and pressure parameters of mechanical ventilation was analyzed. Based on the follow-up of 28-d survival, their Pplat, ΔP, Pmean and APACHE-II scores were compared. The value of APACHE-II score and pressure parameters in the prognostic evaluation of ARDS patients was analyzed.

**Results:**

Pplat, ΔP and Pmean in the low score group were significantly lower than those in the high score group(*P*<0.05). Pplat, ΔP, Pmean and APACHE-II score in the survival group were significantly lower than those in the control group(*P*<0.05). APACHE-II score showed significantly positive correlations with Pplat, ΔP and Pmean. The AUC of Pmean, Pplat, ΔP and APACHE-II score in predicting the prognosis and diagnosis of ARDS patients was 0.761, 0.833, 0.754 and 0.832, respectively.

**Conclusion:**

APACHE-II score of ARDS patients shows significantly positive correlations with pressure parameters of mechanical ventilation, and has diagnostic value for the prognosis of ARDS patients.

## INTRODUCTION

Acute respiratory distress syndrome (ARDS) is a common acute critical disease in ICU, with a mortality of about 40%.^1^ In addition to cardiogenic factors, a variety of pulmonary pathogenic factors can lead to progressive and hypoxic respiratory failure within one week.^2^ Oxygenation index (OI), a traditional index for evaluating the condition of ARDS, has gradually revealed its limitations, but its effect in improving the prognosis is not ideal. The reason may be that OI cannot fully reflect ventilator support and evaluate the risk of ventilator-related lung injury.^3^ Acute physiology and chronic health evaluation (APACHE-II) score is an evaluation system for predicting disease severity and death risk, which is widely used in the diagnosis and treatment of critical patients.^4^ In recent years, studies^5^-^8^ have shown that the pressure parameters of mechanical ventilation [plateau pressure (Pplat), driving pressure (ΔP) and mean airway pressure (Pmean)] have been confirmed to be correlated with the prognosis of ARDS patients. They are the basic parameters of respiratory mechanics, which can be directly measured by ventilator and obtained conveniently, and are important indexes for managing lung protection ventilation strategy in patients with mechanical ventilation. This study has investigated the correlations between APACHE-II score and pressure parameters of mechanical ventilation in patients with ARDS and their value in prognostic evaluation.

## METHODS

This was a retrospective study. Based on the inclusion and exclusion criteria, the clinical data of 79 patients with ARDS treated in Shengzhou Hospital of Traditional Chinese Medicine from April 2020 to April 2022 were analyzed retrospectively. According to whether their APACHE-II scores were higher than 15, they were divided into a low score group (n = 20) and a high score group (n= 59).

### Ethical Approval

The study was approved by the Institutional Ethics Committee of Shengzhou Hospital of Traditional Chinese Medicine (Ref No.: 2023013; dated Jan. 18, 2023), and written informed consent was obtained from all participants. Comparison in general data showed no statistical significance in general data (*P* > 0.05), suggesting comparability (Table-I).

**Table-I:** Clinical data in two groups [(n, %), *χ̅* ±*S*].

Group	N	Age (years)	Gender (n)		BMIkg/m2	Smoking story (N)	Pulmonary vascular resistance index (mL/kg)

			Male	Female			
Low score group	20	58.93 ± 15.49	13 (65)	7 (35)	23.58 ± 3.12	9 (45)	330.12 ± 39.47
High score group	59	60.15 ± 14.37	39 (66.1)	20 (33.9)	22.97 ± 4.14	23 (38.98)	327.51 ± 33.91
		0.322	0.008		0.602	0.224	0.285
		0.749	0.928		0.549	0.636	0.776

### Inclusion criteria:

- ARDS diagnosed by imaging combined with medical history, symptoms and signs.
- Age > 18 and ≤ 75 years.
- No immunodeficiency disease, interstitial lung disease, chronic obstructive pulmonary disease or multiple organ failure.
- Receiving invasive mechanical ventilation.
- Good compliance of the patient and family members and complete clinical data.

### Exclusion criteria:

- Hospital treatment or EICU < 48 h.
- Refusal to cooperate or give up treatment halfway.
- Accompanied by malignant tumors or other diseases that seriously affect the quality of life.
- Recent immunosuppressive therapy.

### Methods

The APACHE-II score of all patients were recorded based on items A, B and C. A: acute physiology score, including 13 indexes such as respiration, heart rate and average blood pressure. Each item was scored according to the degree of deviation from the normal value. The worst value within 24 h after admission was considered as the study score. B: age score, which was divided into five categories: < 45 years old, 0; ≥ 45 years old and < 55 years old, 2; ≥ 55 years old and < 65 years old, 3; ≥ 65 years old and < 75 years old, 5; ≥ 75 years old, 6. C: chronic health evaluation score, which was based on the patients’ previous health status, postoperative complications and surgical method. The total score was the sum of A, B and C, with a maximum of 71. The higher the score, the severer the condition and the higher the mortality.

All the patients were monitored using a Siemens ventilator (MAQUET SERVO-i V3.2), and Pmean, Pplat and ΔP at EICU were recorded.

### Statistical Analysis

The data were analyzed using SPSS22.0. The enumeration data were expressed as n (%) and analyzed by the χ² test. The measurement data were expressed as (*χ̅*±*S*) and analyzed with the *t* test. The correlations between APACHE-II score and pressure parameters of mechanical ventilation were analyzed using Pearson’s correlation analysis. The predictive value of APACHE-II scores and pressure parameters of mechanical ventilation in the prognosis of ARDS patients was analyzed using ROC curve. *P* < 0.05 was considered statistically significant.

## RESULTS

In the low score group (APACHE-II score ≤ 15), Pmean (7.52 ± 0.89), Pplat (16.57 ± 4.39) and ΔP (12.70 ± 4.24) were all significantly lower than those (13.87 ± 4.12, 22.99 ± 5.17 and 15.70 ± 4.84) in the high score group (APACHE-II score > 15), with statistically significant differences (t = 6.811, t = 4.973, t = 2.467, *P* < 0.05), as seen in Table-II.

**Table-II:** Pressure parameters of mechanical ventilation between low and high score groups.

Group	N	Pmean (cmH_2_O)	Pplat (cmH_2_O)	ΔP (cmH_2_O)
Low score group (≤ 15)	20	7.52 ± 0.89	16.57 ± 4.39	12.70 ± 4.24
High score group (> 15)	59	13.87 ± 4.12	22.99 ± 5.17	15.70 ± 4.84
*t*		6.811	4.973	2.467
*P*		< 0.001	< 0.001	0.016

***Notes:*** Plateau pressure, Pplat; driving pressure, ΔP; mean airway pressure, Pmean.

The 28-day follow-up results revealed survival in 53 (67.09%) patients and death in 26 (32.91%) patients. The 79 patients with ARDS were divided into survival group (n = 53) and death group (n = 26), and their pressure parameters of mechanical ventilation and APACHE-II score were compared. In the survival group, Pmean (10.46 ± 3.01), Pplat (19.11 ± 4.56), ΔP (13.46 ± 4.12) and APACHE-II score (17.53 ± 4.12) were all significantly lower than those (15.93 ± 4.93, 25.96 ± 5.02, 17.97 ± 4.88 and 26.48 ± 6.78) in the death group, presenting statistically significant differences (t = 6.104, t = 6.069, t = 4.299, t = 7.277, *P* < 0.05) (Table-III).

**Table-III:** Pressure parameters of mechanical ventilation and APACHE-II score between survival group and death group.

Group	N	Pmean	Pplat	ΔP	APACHE-II score
Survival group	53	10.46 ± 3.01	19.11 ± 4.56	13.46 ± 4.12	17.53 ± 4.12
Death group	26	15.93 ± 4.93	25.96 ± 5.02	17.97 ± 4.88	26.48 ± 6.78
*t*		6.104	6.069	4.299	7.277
*P*		< 0.001	< 0.001	< 0.001	< 0.001

***Notes:*** Plateau pressure, Pplat; driving pressure, ΔP; mean airway pressure, Pmean.

Pearson’s correlation analysis demonstrated that APACHE-II score had significantly positive correlations with Pmean, Pplat and ΔP (r = 0.613, r = 0.493, r = 0.271), as shown in Table-IV.

**Table-IV:** Correlation analysis between APACHE-II score and pressure parameters of mechanical ventilation in ARDS patients.

		APACHEII score	Pmean	Pplat	ΔP
APACHE-II score	Pearson correlation	1	0.613**	0.493**	0.271*
	Significant (two-tailed)		0	0	0.016
	N	79	79	79	79
Pmean	Pearson correlation	0.613**	1.000	0.625**	0.419**
	Significant (two-tailed)	0		0	0
	N	79	79	79	79
Pplat	Pearson correlation	0.493**	0.625**	1.000	0.584**
	Significant (two-tailed)	0	0		0
	N	79	79	79	79
ΔP	Pearson correlation	0.271*	0.419**	0.584**	1.000
	Significant (two-tailed)	0.016	0	0	
	N	79	79	79	79

***Notes:***

** When the confidence (two-tailed) is 0.01, the correlation is significant;

* When the confidence (two-tailed) is 0.05, the correlation is significant.

**Fig.1:**
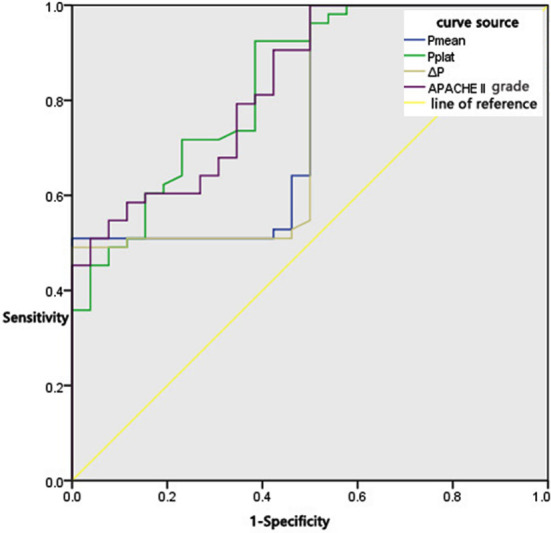
ROC curve.

The predictive value of Pmean, Pplat, ΔP and APACHE-II score in the prognosis of ARDS patients is presented in Table-V and Fig.1. The AUC of Pmean, Pplat, ΔP and APACHE-II score in predicting the prognosis was 0.761, 0.833, 0.754 and 0.832, respectively. The results indicated that ΔP was of high predictive value in the prognosis of patients with ARDS (*P* < 0.05).

**Table-V:** Predictive value of APACHE-II score and pressure parameters of mechanical ventilation in prognosis of ARDS patients.

Variable	AUC	Standard error	P	95%CI	cut-off	Youden index	Sensitivity	Specificity
Pmean	0.761	0.058	0	0.646~0.875	9.605	0.509	50.9	100.0
Pplat	0.833	0.048	0	0.740~0.927	23.830	0.521	90.6	61.5
ΔP	0.754	0.059	0	0.637~0.870	18.800	0.481	98.1	50.0
APACHE-II score	0.832	0.047	0	0.741~0.924	22.295	0.483	90.6	57.7

***Notes:*** Plateau pressure, Pplat; driving pressure, ΔP; mean airway pressure, Pmean.

## DISCUSSION

In this study, the results showed that APACHE-II score of ARDS patients has significantly positive correlations with pressure parameters of mechanical ventilation. Based on 28-day follow-up, it was found that Pmean, Pplat, ΔP and APACHE-II score were lower in the survival group while higher in the death group (*P* < 0.05). Moreover, the predictive and diagnostic value of Pmean, Pplat, ΔP and APACHE-II score in the prognosis of ARDS patients revealed that the sensitivity was 50.9%, 90.6%, 98.1% and 90.6%, respectively, suggesting that they are of high value in the prognostic evaluation of ARDS patients. The combined use of which may achieve a better predictive and evaluation effect in the clinic. The reason may be that the pressure parameters of mechanical ventilation have good bedside and continuous observability^9^, which can provide a more convenient index for predicting the prognosis of ARDS, guiding the clinical management of ARDS and evaluating the efficacy.

ARDS is a common critical disease in emergency, and its pathogenesis is complex. It is characterized by severe pulmonary inflammation, damage to the alveolar epithelium and capillary endothelium, and pulmonary dysfunction.^10^ Additionally, the alveolar fluid clearance and apoptosis are abnormal, which leads to the decline of pulmonary ventilation function.^11^ At present, there is no specific drug to improve the survival of ARDS patients, and the main treatment is general supportive care for severe diseases combined with key mechanical ventilation.^12^,^13^ Therefore, the early prediction of ADRS severity and prognosis may be of great significance to improve the prognosis and reduce the mortality of patients.

Pplat is the pipeline pressure when the patients hold their breath after inhalation and artificially block their breath, which indicates the alveolar pressure at the moment. In this study, Pplat in patients who died within 28 days was significantly higher than that in those who survived within 28-day, which may be related to the increased incidence of ventilation-related lung injury caused by high Pplat. Zhou et al.^14^ pointed out that the decrease of lung compliance in ARDS patients and the use of high-level positive end-expiratory pressure in mechanical ventilation can lead to the increase of Pplat. Meanwhile, with the continuous progress of mechanical ventilation, the airway pressure and Pplat may increase, and the mortality of ARDS patients will increase significantly. Pmean is the mean pressure of the airway during the complete ventilation cycle. Sahetya SK et al.^15^ found that among ARDS patients, patients with higher Pmean have higher mortality. Although Pmean has high sensitivity in evaluating prognosis, it has many influencing factors^16^, so it needs to be used in combination with other indexes. ΔP is the pressure value required to overcome the elastic retraction force of the respiratory system. The elasticity of the respiratory system is related to cross pulmonary elasticity and chest wall elasticity.^17^ Some scholars found that when ΔP increases by seven cm H_2_O, the 60-d mortality rate of ARDS patients also increases, and ΔP may be one of the best indexes to evaluate the prognosis of patients with ARDS. However, according to Constantin et al.^18^, ΔP is correlated with both cross pulmonary elasticity and thoracic elasticity, while chest wall edema, pleural effusion and obesity can all affect thoracic elasticity. Thus, the role of ΔP in predicting the prognosis of patients with ARDS needs to be further explored and verified. APACHE-II score is mainly used to evaluate the severity of this disease, which is helpful to evaluate the prognosis of the disease and guide the treatment plan. Furthermore, foreign studies^19^ showed that APACHE-II score is positively correlated with lung injury. Therefore, early monitoring of APACHE-II score in patients with ARDS may more accurately determine the severity and prognosis of lung injury.

### Limitations of this study

This was a retrospective descriptive study, with limited clinical data available and limited persuasive conclusions. Further intervention trials are needed in the future to confirm these results.

## CONCLUSION

In conclusion, APACHE-II score of ARDS patients shows significantly positive correlations with pressure parameters of mechanical ventilation, and has diagnostic value for the prognosis of ARDS patients. In future clinical practice, this can conveniently and rapidly determine the condition of patients with ARDS and predict their mortality, so as to provide a basis for standard and effective treatment of ARDS in the early stage.

### Authors’ Contributions:

**WL and** **JZ** designed this study, prepared this manuscript, are responsible and accountable for the accuracy and integrity of the work.

**YQ and** **NF** collected and analyzed clinical data.

**LY** Data analysis, significantly revised this manuscript.
